# Supplementary material for: Mulberry EIL3 confers salt and drought tolerances and modulates ethylene biosynthetic gene expression
Source: PeerJ. 2019 Feb 19;7:e6391. doi: 10.7717/peerj.6391 (PMC6385683; doi:10.7717/peerj.6391)
Supplement: Supplemental Information 1 [file peerj-07-6391-s001.docx]

**Table S1 The prediction of the EIL-binding sites in the promoter of *MnACO* and *MnACS* genes.**

| Promoter | Length of nucleotides (bp) | PERE binding sites | ECBS binding sites |
| --- | --- | --- | --- |
| *MnACO1* | 1518 | 2 | 1 |
| *MnACO2* | 1429 | 1 | 1 |
| *MnACS1* | 1513 | 2 | 6 |
| *MnACS3* | 1376 | 2 | 1 |
