# Supplementary material for: Mulberry EIL3 confers salt and drought tolerances and modulates ethylene biosynthetic gene expression
Source: PeerJ. 2019 Feb 19;7:e6391. doi: 10.7717/peerj.6391 (PMC6385683; doi:10.7717/peerj.6391)
Supplement: Supplemental Information 2 [file peerj-07-6391-s002.docx]

**Table S2 Primers used in this study.**

| Purpose | Primer name | Sequence (5'-3') |
| --- | --- | --- |
| Plant expression vectors construction | MnEIL1 F | CATGCCATGGTAATGGGCATTTTCGAAGAGCT |
|  | MnEIL1 R | GAAGATCTCCGATGTACCAGAGGGAGACAT |
|  | MnEIL2 F | GAAGATCTGATGGGGTTTTCTGGTAAT |
|  | MnEIL2 R | GGACTAGTGAGGTACCAGAGCGAGACGT |
|  | MnEIL3 F | CATGCCATGGTAATGGCCTATGACTCATTGGT |
|  | MnEIL3 R | GAAGATCTCCTGCTCCGAAGTAATGAATCA |
|  | MnACS1pro F | GTGAGTAGAAATGGTGGATGAC |
|  | MnACS1pro R | TATTAATTTGGTTTTCTCGGATGAG |
|  | MnACS3pro F | CCCATTTGAAACATTGTGGCAC |
|  | MnACS3pro R | CGAACAATATTTTGTTATGATC |
| Expression  pattern  analysis | MnEIL1 F | GGAGGCTTTAGAGCATGAGC |
|  | MnEIL1 R | TCAATAGCATGCGGTCTCTC |
|  | MnEIL2 R | AACGATGATGCTGGATCAGA |
|  | MnEIL2 R | ACGGTATGGGCAATTCATTT |
|  | MnEIL3 F  MnEIL3 R | AACGGGACATCTGGGATAAC  ATCAGTGCCATCAACGTCAT |
|  | MaACS1 F | GCAACACTGACCTCATCCAC |
|  | MaACS1 R | TGCGAGGATACTAATCCGAA |
|  | MaACS3 F | TGGCTTCCAAATCACTGAAG |
|  | MaACS3 R | GGTTGTGTTCGTTCGATGTC |
|  | MaACTIN3 F | GCATGAAGATCAAGGTGGTG |
|  | MaACTIN3 R | CATCTGCTGGAAGGTGCTAA |
|  | AtACS2 F  AtACS2 R | ACCTCTTCTCCGAGCATGAA  GCCGTCAAAAACAACCCTAA |
|  | AtACS3 F  AtACS3 R | ATGTCTCAGGGTGCATGTGA  GGCGAGACCCATTTGAATAA |
|  | AtACS4 F  AtACS4 R | AATGTCAAGTTTCGGCCTTG  GATGGCCTCTAGACCCAACA |
|  | AtACS6 F  AtACS6 R | GACGAGTTTATCCGCGAGAG  ACACGCCATAGTTCGGTTTC |
|  | AtACS7 F  AtACS7 R | GATGGAGAACCGGAGTGAAA  GGTTCGATGGGTTGGTTATG |
|  | AtACS8 F  AtACS8 R | GAGAACCGGAGCTGAGATTG  GTAGTGCCCAACGGGTTAGA |
|  | AtACS10 F  AtACS10 R | GGCGAACAGGAGTTGACATT’  TGGGATTTGAAGGATTCGAG |
|  | AtACS12 F  AtACS12 R | AGAACGAAGCTCCCACAAGA  GTTTCGATATCCGGTGCTGT |
|  | AtACO1 F  AtACO1 R | GAATGCCTTTTCTGGTCCAA  CTTGGATGGCGGTATAGGAA |
|  | AtACO2 F  AtACO2 R | TGGTGACCAACTTGAGGTGA  CGGAATCTTTCTCGACAAGC |
|  | AtACO4 F  AtACO4 R | ATAATCCGGGAAGCGACTCT  CATTGTTGGCCACAGTTGTC |
|  | β-actin2 F  β-actin2 R | TGCTGAGCTTATCGATTCCG  TTCGGTGATGGGAATACAG |
| Inverse-PCR | Reverse F1 | TATGGAGAAACTCGAGCTTGTC |
|  | Reverse R1 | GATCCCCCGAATTAATTCGGCG |
|  | Reverse F2 | AGATCCGGTCGGCATCTACTCT |
|  | Reverse R2 | AGCGTCAATTTGTTTACACCAC |
