# Supplementary figures and images for: Mulberry EIL3 confers salt and drought tolerances and modulates ethylene biosynthetic gene expression
Source: PeerJ. 2019 Feb 19;7:e6391. doi: 10.7717/peerj.6391 (PMC6385683; doi:10.7717/peerj.6391)

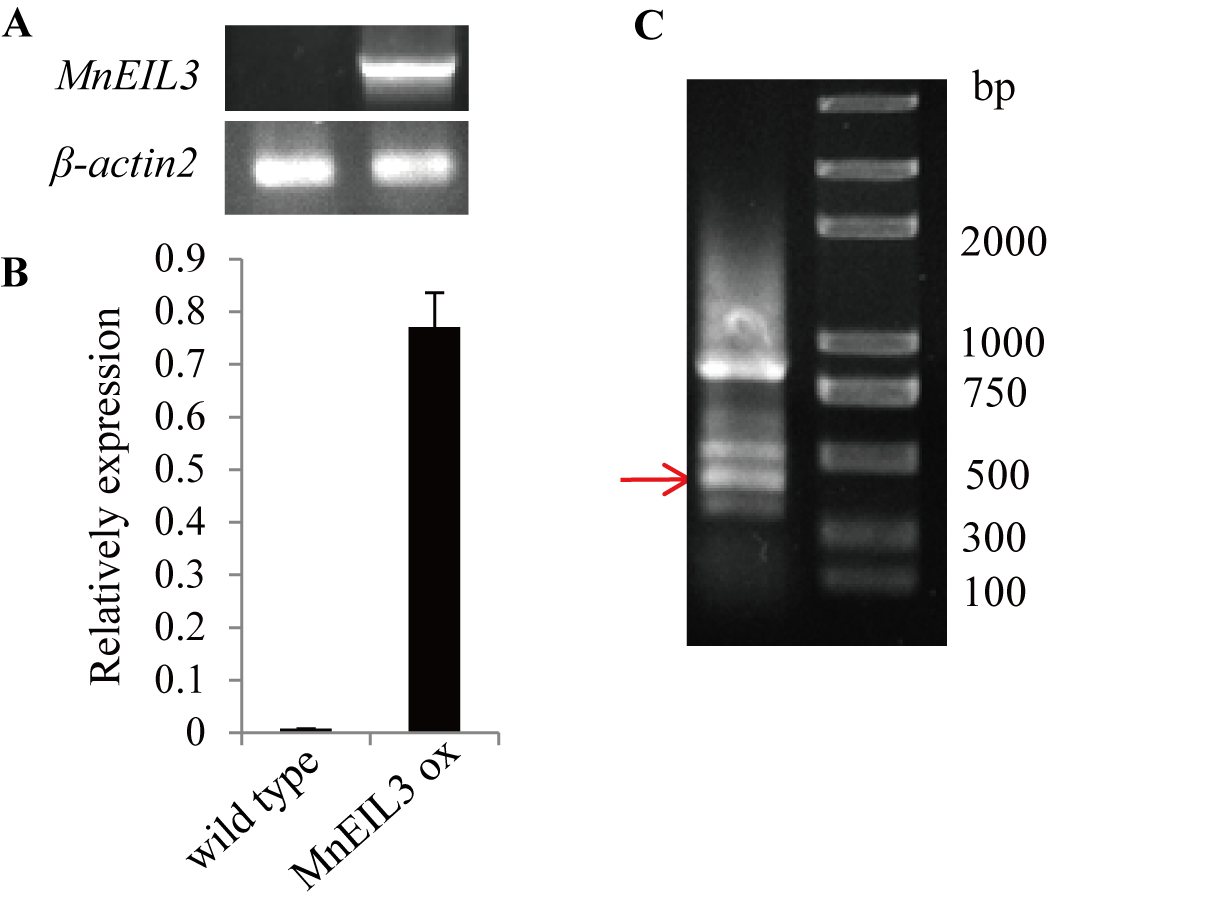

Supplement: Supplemental Information 3 — (A) Genomic PCR analysis of transgenic lines. (B) Quantitative real-time PCR. (C) Inverse PCR. [file peerj-07-6391-s003.png]

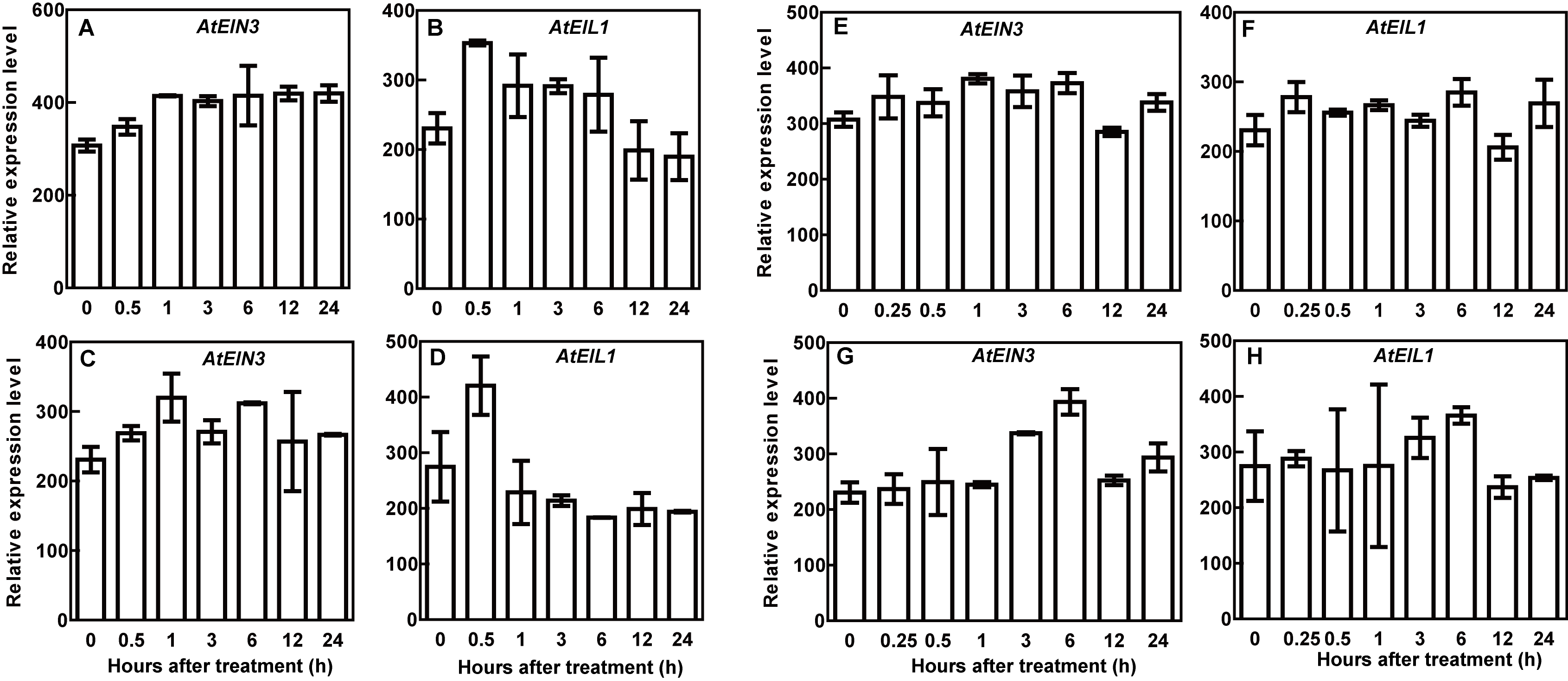

Supplement: Supplemental Information 4 — (A–B) AtEIN3 and AtEIL1 genes’ expression in roots under salt stress. (C–D) AtEIN3 and AtEIL1 genes’ expression in shoot under salt stress. (E–F) AtEIN3 and AtEIL1 genes’ expression in roots under drought stress. (G–H) AtEIN3 and AtEIL1 genes’ expression in shoot under drought stress. These data were downloaded from the ePlant database (http://bar.utoronto.ca/eplant/). [file peerj-07-6391-s004.png]
